# Mitochondrial dysfunction in aging: Much progress but many unresolved questions

**DOI:** 10.1016/j.bbabio.2015.05.022

**Published:** 2015-11

**Authors:** Brendan A.I. Payne, Patrick F. Chinnery

**Affiliations:** Wellcome Trust Centre for Mitochondrial Research, Institute of Genetic Medicine, Newcastle University, UK

**Keywords:** DNA, Mitochondrial, Aging

## Abstract

The free radical theory of aging is almost 60 years old. As mitochondria are the principle source of intracellular reactive oxygen species (ROS), this hypothesis suggested a central role for the mitochondrion in normal mammalian aging. In recent years, however, much work has questioned the importance of mitochondrial ROS in driving aging. Conversely new evidence points to other facets of mitochondrial dysfunction which may nevertheless suggest the mitochondrion retains a critical role at the center of a complex web of processes leading to cellular and organismal aging.

## Highlights

• Mitochondrial dysfunction retains a central position in aging theory.
• Clonal expansion of mtDNA mutations occurs in aging post-mitotic tissues.
• ROS may have both deleterious and beneficial roles.

## 1 Introduction

Understanding the basis of human aging such that we might ultimately slow its course is one of the great biomedical challenges for the 21st century. Age is the most important risk factor for most of the common diseases. Although our knowledge of the aging process remains far from complete, most biogerontologists would now agree that aging starts with molecular damage, leading to cell, tissue and ultimately organ dysfunction [1,2]. This intrinsic aging process is seen as forming a ‘tapestry’ upon which the diseases of older age may appear. The opposing views would be that aging is simply the net result of accumulating chronic diseases, or that aging and chronic disease are parallel but unrelated processes. Perhaps the best known and most long-standing hypothesis to explain aging is the free radical theory, which proposes a central role for the mitochondrion as the principle source of intracellular reactive oxygen species (ROS) leading to mitochondrial DNA (mtDNA) mutations [3]. Somatic (acquired) mtDNA mutations have been extensively reported in normal human aging, particularly in post-mitotic tissue such as skeletal muscle and neurons, but also in replicative tissue such as the colonic crypt, and somatic mtDNA mutations are also well-described in age-associated neurodegenerative diseases [4–16]. Corresponding declines in mitochondrial function with age are also well described. However, these observations do not necessarily imply a causal relationship between mitochondrial dysfunction and human aging. In recent years the mitochondrion has once against assumed a pre-eminent role in aging research, driven in part by the development of an important mouse model [17,18]. Ironically, much of the recent work has cast doubt on the mitochondrial free radical theory of aging, but at the same time, important steps forward have been made in better understanding the nature of mitochondrial aging. Particularly important amongst these advances have been an increased awareness of the origin and natural history of mitochondrial mtDNA mutations in aging, and an increased ability to link the mitochondrion with other cellular pathways of aging. As a result we are now arriving at a more nuanced and complex understanding of mitochondrial aging, which will hopefully offer a better chance of effective intervention over the next decades. Nevertheless there remain a number of unresolved controversies and contradictory observations within the field. As such in this introductory review we will consider some recent advances in the field, framed here as a number of the more important unresolved questions.

### 1.1 Mitochondrial DNA mutations and aging: oxidative damage or replication error?

Mitochondria are ubiquitous intracellular organelles, present in almost all mammalian cells. Their primary role is of adenosine triphosphaste (ATP, the main source of intracellular energy) production through oxidative phosphorylation. Mitochondria contain their own small 16.5 kb circular chromosome of DNA encoding several key proteins of the mitochondrial respiratory chain [19]. However the majority of the > 1000 predicted mitochondrially targeted proteins are encoded by the nuclear genome. The mitochondrial respiratory chain comprises 5 multi-subunit complexes, the last of which being ATP synthase. Electrons are exchanged down the chain at increasing reduction potentials from complexes I through IV, allowing the shuttling of protons across the mitochondrial membrane creating a proton gradient (membrane potential). Proton flux through the ATP synthase then provides the energy which drives ATP synthesis. Some premature electron leak inevitably occurs at the respiratory chain, resulting in the generation of superoxide radicals. Specifically, complexes I and III are reported to be the major sources of ROS [20]. Partial uncoupling (inefficiency) of the respiratory chain allows some proton leak, that is, movement of protons back into the mitochondrial matrix space that does not occur *via* ATP synthase. This makes the respiratory chain less efficient, and physiologically is used for thermogenesis in brown fat. It has been previously assumed that uncoupling might result in increased oxidative damage. States of marked uncoupling are highly deleterious and are associated with increased ROS. However, mild uncoupling in fact significantly reduces ROS production. It has been suggested (albeit controversially) that mtDNA subhaplogroups associated with mild uncoupling may have been selected for their increased thermogenesis in cold climates [21], but may also confer a longevity advantage due to decreased ROS. The mutation rate of the mitochondrial genome is estimated to be ~ 15 × that of the nuclear genome. This observation arises from several considerations: 1) the mitochondrial genome is located on the inner mitochondrial membrane, adjacent to the respiratory chain, which is the major source of intracellular ROS production; 2) the mitochondrial genome lacks protective histones; 3) the DNA repair mechanisms are limited compared with the nuclear genome. It was therefore long assumed that ROS was the major source of somatic (acquired) mtDNA mutations in aging [22,23]. The mitochondrial theory of aging goes on to postulate that the accumulation of mtDNA mutations will lead to abnormalities of mitochondrial respiratory chain proteins, causing partial uncoupling of the respiratory chain. This in turn will lead to further increased ROS and more mtDNA mutations. Such a ‘vicious cycle’ hypothesis would predict an exponential rather than linear trajectory of increasing mtDNA mutation burden, as the initial mutations would provoke a further mutational ‘burst’. In fact, however, recent studies suggest that mtDNA mutational burden may not significantly increase at all during human aging, suggesting that a model based on ROS does not properly explain the natural history of mtDNA mutations over the human life-course [24,25].

In contrast, recent data have suggested an importance for naturally occurring replication errors in the formation of age-associated mtDNA mutations. The characteristic mtDNA mutation type in post-mitotic tissues (such as muscle and neurons) is the large-scale deletion [26]. Such mutations typically delete several kbs of the mitochondrial genome, and as this is composed almost entirely of coding genes, such mutations are highly likely to have a functional effect. Large-scale deletions have a very characteristic distribution within the ‘major arc’ of the mitochondrial genome, between the origins of replication. The 5′ and 3′ ends of the deletion are clustered around hotspots associated with homologous repeats [27–29]. The classic example is the 4977 bp ‘common deletion’ which is associated with 13 bp homologous repeats at each end. The majority of deletions are similarly associated with homologous (or near homologous) repeats. Recent physicochemical modeling suggests that once formed these deleted mtDNA species have inherent stability [27]. The importance of homologous repeats in deletion formation suggests a role for single-stranded DNA (ssDNA) intermediates as these will allow the homologous repeats to anneal. Previously this phenomenon had been thought to arise through the ‘strand asynchronous’ mechanism of mtDNA replication. More recent data suggest however that double-stranded breaks (DSBs) may be the driving force [30]. These could arise through a variety of processes known to occur naturally including: replication stalling, oxidative damage and UV radiation. Once a DSB has formed, repair of the mtDNA molecule will be attempted by exonuclease activity which initially creates ssDNA. This can then anneal at homologous repeats, leading to the mtDNA deletion. This recent hypothesis however remains controversial and many authors remain in favor of the previous model of slipped mispairing [31].

### 1.2 Mitochondrial aging and the ‘mutator’ mouse: proof of causality?

About a decade ago, two very similar mouse models were developed almost simultaneously which have revealed many new insights into mitochondrial aging [17,18]. These mice have a homozygous knock-in mutation (*Polg^D257A/D275A^*) for an error-prone polymerase gamma (the sole mtDNA polymerase). These mice are referred to as *PolgA*, or colloquially as the ‘mutator mice’. They show greatly increased accumulation of somatic mtDNA mutations throughout life, associated with significantly reduced longevity, and a marked progeroid phenotype that recapitulates the vast majority of phenotypic features of normal human aging including: kyphosis, reduced fertility, testicular atrophy, cardiomyopathy, hemopoietic stem cell decline, and frailty.

Prior to the development of the ‘mutator’ mouse the evidence for a role of mtDNA mutations in aging was largely correlative. That is, although a number of studies had reported somatic mtDNA mutations in aged persons (as described above), it was possible that these were simply a marker of chronological rather than biological age. The mouse models appeared to suggest that mtDNA mutations had a causal role in aging. Closer scrutiny, however, revealed that the true picture was likely to be more complex. Although the homozygous mouse has a clear progeroid phenotype, this is associated with a vastly increased mtDNA mutation rate. The heterozygous mouse has a modestly increased mutation rate, which appears to exceed that seen in an elderly human, but has an apparently normal phenotype [32]. These further observations led some authors to suggest that the ‘mutator’ mouse could not properly capitulate mtDNA mutations in normal human aging. Whilst this objection has some currency, the model should not however be rejected out of hand [33]. A key further consideration is the great difference in lifespan between humans (> 80 years) and mice (~ 3 years). MtDNA is constantly turned over throughout life, even in non-dividing cells, and to the best of our knowledge the rate of turnover (‘half-life’) of mtDNA is likely to be very similar in mice and humans. Therefore, the elderly human has experienced vastly more cycles of mtDNA replication than the aged mouse. Recent data suggest that cycles of mtDNA replication are likely to play a critical role in the natural history and functional relevance of mtDNA mutations in aging, as is discussed in the following section.

Finally there is some controversy over the types of mutations seen in the ‘mutator’ mouse, the extent to which these reflect those seen in normal human aging, and which type(s) may drive the phenotype. Linear forms of mtDNA (which are presumably not being properly degraded) seem to be particularly common in the mouse model but are not thought to be an important feature of normal human aging. In contrast ‘canonical’ deletions occur rather rarely if at all in the ‘mutator’ mouse [34,35].

### 1.3 Clonal expansion: the importance of early mutations?

Normal mammalian cells contain multiple copies of the mitochondrial genome, typically hundreds to tens of thousands per cell. Thus any mtDNA mutation will co-exist with the wild-type within a cell, a state known as heteroplasmy. Typically the mutant mtDNA must exceed a heteroplasmy level of ~ 70% in order to cause a functional defect (although this may vary somewhat between mutation types) [36,37]. A somatic mutation will presumably initially exist as a unique species within a cell. How can it therefore reach a sufficient heteroplasmy level to cause a functional defect? This process is known as clonal expansion, and broadly speaking could either occur selectively (*i.e.* the mutant mtDNA species expands preferentially at the expense of the wild-type), or neutrally. A selective expansion, based on differential size, is plausible for large-scale deletion mutations, and there is some *in vitro* evidence to support its occurrence [38]. A neutral theory of clonal expansion is based simply on the notion that mtDNA is continuously turned over in non-dividing cells (termed ‘relaxed replication’) [39–41]. By chance, in a minority of cells a mutant mtDNA species will increase to a significant level through random drift. This process was predicted to be slow (progressing over decades), and thus implied a functional importance for mutations arising early in life [42].

Several strands of recent evidence have renewed interest in the importance of early mutations and the consequent effects of clonal expansion. Firstly as discussed above, a key difference between an elderly human and the mouse model is the number of cycles of mtDNA replication. As mtDNA replication is the ‘engine’ of clonal expansion, very modest initial mutation burdens may nevertheless be sufficient to ultimately lead to functional defects in elderly humans given the very long period available for clonal expansion. Secondly, and as alluded to earlier, a few recent experiments (including our own) using a variety of modern techniques to measure the total mutational spectrum in tissue homogenates suggest that there may not be a significant increase with aging in humans [24,25]. A larger experiment would however be required to properly answer this question. Although these observations seem at first paradoxical, the key point is that the number of cells containing clonally expanded mtDNA mutations does however increase very significantly with age. These data imply that the natural history of mtDNA mutations in human aging is characterized by the progressive clonal expansion of a limited pool of early mutations, rather than by continuous mutagenesis. This notion again argues against the importance of ROS in driving the accumulation of mtDNA mutations in aging. In an analogous experiment, we have recently shown that long-term HIV-infected patients (who show certain features of an accelerated aging phenotype) have an anti-retroviral drug-induced increase in clonally expanded mtDNA mutations, but not overall mutational burden [43]. Finally, if early mutations are important, then what if the low-level ‘seeding’ mutations are actually not somatic at all, but maternally inherited? We have studied pairs of related and unrelated individuals using massively parallel deep resequencing, and showed that many ostensibly somatic mtDNA mutations are in fact transmitted down the maternal line [25]. This observation has recently been nicely complemented by further work on the ‘mutator’ mouse. It was observed that the ‘wild-type’ offspring of a heterozygous ‘mutator’ mother will have germline mtDNA mutations. These mice show a mildly progeroid phenotype despite normal polymerase gamma function [44].

### 1.4 ROS: how much is too much?

**Fig. 1:**
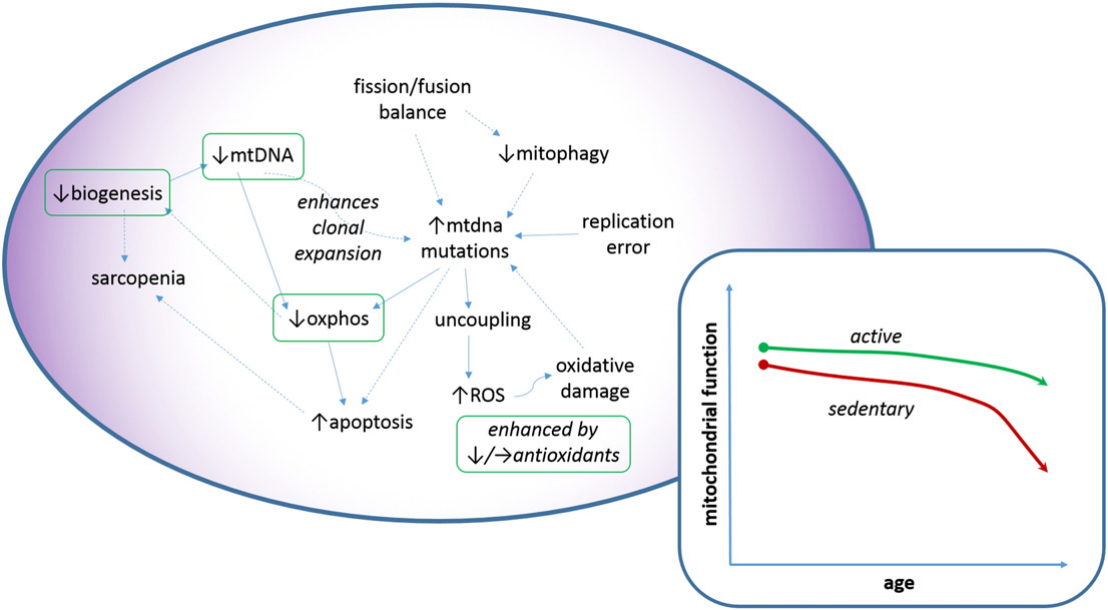
The sedentary elderly mitochondrion. Schematic shows key mitochondrial changes in a sedentary elderly individual. Solid arrows indicate likely casual relationships, whereas dashed arrows are more speculative relationships. Green boxes indicate those processes which are likely to be subject to improvement by endurance exercise or increased physical activity. Panel insert shows the expected decline in mitochondrial capacity with age in active and sedentary individuals.

We have discussed in detail why ROS may no longer be considered as the major playing in driving age-associated mtDNA mutation. Nevertheless it remains likely that mitochondrial ROS production causes other cellular damage that is plausibly causally associated with the aging process. Taken together, most data suggest that there is an increase in oxidative damage in elderly humans [45–47]. Some papers suggest that antioxidant defenses decrease with age, whereas many suggest that they remain essentially unchanged [48,49]. It certainly seems likely that antioxidant defenses probably do not make a compensatory increase to deal with increased oxidative stress. Calorie restriction (CR) is the intervention for which the greatest evidence exists for slowing aging in a variety of model organisms. It was initially thought that CR would lead to lowered basal metabolic rate (BMR) and hence decreased ROS production. In fact CR actually can lead to increased BMR by triggering mitochondrial biogenesis, but the ability to scavenge ROS increases (in a SIRT3 dependent manner) [50]. Likewise the very long-lived rodent, the naked mole rat, actually shows increased ROS compared with other rodents, but has increased free radical scavenging, which does not decline with age. In a very similar vein, endurance exercise (the other key modality which appears to slow mitochondrial aging), causes an initial rise in ROS, but this is more than compensated by the stimulation of biogenesis and an increase in free radical scavenging [51,52]. It now appears that intracellular ROS may also play some important roles in intracellular signaling that may actually impact beneficially on the aging process [53,54]. We are thus coming to a modern nuanced view that all ROS is not necessarily bad, and that it is the balance of ROS and scavenging that is probably key, along with the subcellular location in which the ROS is acting (Fig. 1).

If ROS are important in aging, we would expect supplemental antioxidants to be beneficial. In fact in human studies they have no beneficial effects and may even be harmful [55]. The problem may be one of targeting to the mitochondrion. What can transgenic rodents therefore tell us about the role of ROS in aging? Several models have tried to determine whether over-expression of an antioxidant is helpful. Results have been mixed, with one model where the antioxidant expression was not mitochondrially-targeted showing no effect, whereas a targeted antioxidant, such as catalase, appeared to result in an attenuated rate of aging [56,57]. Conversely a mitochondrial antioxidant-deficient mouse has a very adverse phenotype with premature death due to mitochondrial dysfunction and neurodegeneration [58]. In the ‘mutator’ mouse ROS production and oxidative damage are generally not significantly increased, suggesting that ROS may not be a necessary requirement for expression of the progeroid phenotype (although see later discussion regarding stem cell aging) [18,59].

### 1.5 Mitochondrial function in aging: does phenotype follow genotype?

As discussed above, somatic mtDNA mutations will initially exist at very low heteroplasmy levels. Even considering all mutations present in a tissue homogenate from an elderly human, they will only represent a relatively small proportion of the total mtDNA pool. At first sight, it is therefore hard to imagine how such mutations would be sufficient to cause a functional effect that could drive an aging phenotype. This has been one of the arguments against a causal role for mtDNA mutations in normal human aging. Again however, clonal expansion is likely to be of critical importance. For example, if 10% of mtDNA molecules in a sample of skeletal muscle tissue are mutated but these are spread evenly throughout all the cells, then we would expect a negligible functional impact as all cells will contain 90% wild-type mtDNA. However, in reality an elderly individual will have 90% of cells which contain almost entirely wild-type mtDNA, and 10% of cells containing very high levels of clonally expanded mtDNA mutations. In the latter case, 10% of cells will experience a functional defect, which could very reasonably lead to tissue dysfunction. In practice such cells containing clonally expanded mtDNA mutations may be identified by sequential cytochrome c oxidase/succinate dehydrogenase (COX/SDH) histochemistry. COX contains subunits encoded by the mitochondrial genome. In the event of an mtDNA defect (present at a high enough level within the cell) COX activity will be lost. Counter-staining with SDH will be retained as this is encoded entirely by the nuclear genome. COX/SDH histochemistry therefore gives one simple measure of mitochondrial function, and proportional COX defects are seen to progressively increase with age in a number of post-mitotic tissues in elderly humans, as well as some replicative tissues such as the colonic crypt [60–64].

When respiratory chain complex activities are measured in the tissue homogenate, the associations with aging are rather mixed. Overall it seems that complex IV activity will often decrease (in keeping with the observed histochemical COX defects), but other complex activities may be retained. Certainly levels of mitochondrial proteins do not appear to decline during human aging, and it is likely that decreases in respiratory chain activity are related to decreased efficiency. Again, this could imply an importance for mtDNA mutations. The end result of the activity of the respiratory chain complexes should be ATP flux. This can be determine *ex vivo* [65], or measured *in vivo* by phosphorus magnetic resonance spectroscopy (^31^P MRS) of skeletal muscle [66]. Again data on changes with aging are mixed but it has been suggested that there is an average decline of 8% per decade in ATP producing capacity [67–69]. Such decreases in mitochondrial function, as well as mitochondrial gene expression are also observed in mice, but not in the very long-lived naked mole rat [70,71]. If cells from an elderly individual have less ATP production then presumably they must either be able to adapt to function with less ATP or they may produce more energy through non-oxidative metabolism [72]. This change may result in knock-on disadvantageous changes in the cell, potentially affecting other signaling pathways associated with aging/longevity.

### 1.6 Mitochondrial biogenesis, aging and decreased physical activity: chicken or egg?

To a certain extent, mtDNA copy number, mitochondrial content and mitochondrial function are correlated. In aging, skeletal muscle mass decreases from mid-life onwards. The annual rate of loss is said to be almost 1%, although the trajectory probably gradually increases with advancing age [73,74]. Along with this loss of mass, is a reduction in mitochondrial functional capacity, but the decrease in mitochondrial capacity is out of proportion, such that the mitochondrial density also decreases [75,76]. In a similar way muscle strength per given muscle mass is also seen to fall with age [77]. However it is unclear to what extent such changes are due to aging *per se*, as opposed to inactivity. It is now accepted that many studies showing apparent deterioration in mitochondrial function or content with age may be heavily confounded by decline in activity with age. In studies where subjects have been stratified by activity levels it is interesting to note that the age-related declines are seen in the sedentary group [78–81]. The active group shows a very mild decline only. When scrutinized even further however there is also the suggestion that sedentary young and elderly are not equivalent in their activity levels. Although neither group is doing regular exercise, the sedentary young may perform more activity during day-to-day living. There may therefore be some useful role in the elderly for increasing their ‘normal’ activities rather than focusing on exercise. Despite the clear links between inactivity and aging, these observations could nevertheless be consistent either with a view that inactivity is the primary driver of mitochondrial decline with age, or that mitochondrial decline is the initial event, and inactivity then serves as a signal amplifier.

Clearly there are therefore very strong links between lack of activity/endurance exercise and age-related mitochondrial decline. A seminal paper recently showed that the progeroid phenotype in the ‘mutator’ mouse could be essentially entirely ameliorated by endurance exercise [82,83]. The ways in which exercise ameliorates mitochondrial function are likely to be complex and involve several cellular pathways, however an AMP kinase/PGC1α pathway mediated increase in mitochondrial biogenesis is thought to play a key role. Certainly when elderly subjects are subjected to exercise training a significant improvement in mitochondrial function can be achieved along with an increase in PGC1α expression [84,85]. In further support of this notion, over-expression of PGC1α improves mitochondrial function [86]. Interestingly however, the ability for AMPK-mediated stimulation of biogenesis does appear to decrease in old age, suggesting that some elderly persons may have mitochondria that are relatively ‘exercise resistant’ [87].

### 1.7 Fission and fusion: mitochondrial damage limitation?

In recent years it has become increasingly recognized that mitochondria do not exist as isolated organelles, but rather they form into complex dynamic networks within cells. The processes of mitochondrial fission and fusion govern the architecture of these networks. The balance of fission and fusion may play an important role in controlling the expression of deleterious mtDNA mutations within cells. On the one hand mitochondrial fusion may allow ‘dilution’ of a mutant mtDNA species into a pool of wild-type molecules, thus reducing the functional effect. On the other hand fission may allow the segregation of abnormal mitochondria within the cell which can then be the subject of selective mitophagy [88–91]. Currently it is not very clear to what extent there may be age-associated changes in mitochondrial fission and fusion or to what extent any such changes might be deleterious or adaptive. A study has reported that Mfn2 expression (a key mediator of mitochondrial fusion) is reduced in aged persons [92]. There is a suggestion of some decrease in mitophagy with aging, which may contribute to the accumulation of mutant mtDNA [93–95].

### 1.8 Sarcopenia: is it mitochondrially mediated?

Sarcopenia is the classical skeletal muscle phenotype of aging and has been associated with the adverse physical state of frailty [96,97]. Sarcopenia is often measured by various imaging modalities demonstrating an age-associated reduction in muscle mass. Age-associated loss of muscle mass and the correlation with mitochondrial function has already been described above. Plausibly this process might at least in part be explained by loss of muscle fibers through apoptosis [98,99]. Mitochondria are clearly intimately linked with apoptosis, and an increase in caspase-independent apoptosis with age is well described, both in skeletal muscle and more generally [97,100–102]. In contrast, caspase-dependent apoptosis does not appear to be increased in aging [102]. Several of these studies have been able to draw broad links between markers of apoptosis, sarcopenia and mitochondrial function.

In contrast however, the presence of sarcopenia has if anything been more closely linked with other systemic features of aging, such as inflammation, than it has with mitochondrial dysfunction. For example, a gene expression study of sarcopenia showed signals from inflammatory and apoptotic pathways without a clear mitochondrial signal [103].

### 1.9 A role for mitochondria in replicative senescence?

Stem cell senescence is considered an important facet of biological aging. Recent work has investigated whether mitochondrial aging might also contribute to stem cell aging. Interestingly, the initial descriptions of the ‘mutator’ mouse suggested that ROS was not increased, however further work tells us that the mouse exhibits stem cell aging (both hemopoietic and neural) [104]. This occurs very early (during fetal development) and therefore precedes both the onset of the progeroid phenotype and the measureable mitochondrial functional defects. The phenomenon may therefore be driven by an early increase in ROS within the stem cells, particularly as antioxidant treatment was seen to ameliorate the stem cell defect.

Recently links have been made between mitochondrial aging and other cellular pathways that are thought to play a key role in aging including IGF-1 signaling and mTOR pathways [105,106]. These pathways have upstream effects on mitochondrial metabolism, and also mediate some of the longevity benefits of CR. Finally, links are now emerging between mitochondrial function, telomere shortening and the p53 pathways, thus joining many of the remaining dots of cellular aging [107].

## 2 Conclusions

It is clear that mitochondria retain a central role in the complex balance of cellular processes that may ultimately contribute to aging. The articles in this themed issue will explore various aspects of this role in much greater detail. A key challenge for the next 10 years will be to clinically translate the novel observations from model systems.

## Conflict of interest statement

We confirm that both authors have no relevant conflicts of interest to declare.
